# Piperine induces autophagy by enhancing protein phosphotase 2A activity in a rotenone-induced Parkinson's disease model

**DOI:** 10.18632/oncotarget.11661

**Published:** 2016-08-27

**Authors:** Jia Liu, Min Chen, Xue Wang, Yi Wang, Chunli Duan, Ge Gao, Lingling Lu, Xia Wu, Xiaomin Wang, Hui Yang

**Affiliations:** ^1^ Department of Neurobiology, Capital Medical University, Center for Parkinson's Disease, Beijing Institute for Brain Disorders, Key Laboratory for Neurodegenerative Diseases of the Ministry of Education, Beijing, China; ^2^ Department of Clinical Laboratory, China Rehabilitation Research Center, School of Rehabilitation Medicine, Capital Medical University, Beijing, China; ^3^ School of Traditional Chinese Medicine, Capital Medical University, Beijing, China

**Keywords:** piperine, Parkinson's disease, autophagy, PP2A, treatment, Gerotarget

## Abstract

Parkinson's disease (PD) is the second most common neurodegenerative disorder, but there are few treatments currently available. The autophagy pathway plays an important role in the pathogenesis of PD; modulating this pathway is considered to be a promising treatment strategy. Piperine (PIP) is a Chinese medicine with anti-inflammatory and antioxidant effects. The present study investigated the neuroprotective effects of PIP on rotenone-induced neurotoxicity in SK-N-SH cells, primary rat cortical neurons, and in a mouse model. Mice were administered rotenone (10mg/kg) for 6 weeks; PIP (25mg/kg, 50mg/kg) was subsequently administered for 4 weeks. We found that PIP treatment attenuated rotenone-induced motor deficits, and rescued the loss of dopaminergic neurons in the substantia nigra. PIP increased cell viability and restored mitochondrial functioning in SK-N-SH cells and primary neurons. In addition, PIP induced autophagy by inhibiting mammalian target of rapamycin complex 1(mTORC1) via activation of protein phosphotase 2A (PP2A). However, inhibiting PP2A activity with okadaic acid reduced these protective effects, suggesting that PP2A is a target of PIP. These findings demonstrate that PIP exerts neuroprotective effects in PD models via induction of autophagy, and may be an effective agent for PD treatment.

## INTRODUCTION

Parkinson's disease (PD) is the second most common neurodegenerative disease after Alzheimer's disease (AD) and is characterized by the progressive loss of dopaminergic neurons of the substantia nigra pars compacta and the deposition of intraneuronal inclusions known as Lewy bodies [1]. Although the pathological features of PD have been extensively described, its etiology remains obscure [2].

Mitochondrial dysfunction has been shown to contribute to PD. Rotenone, which is widely used to model PD [3, 4], disrupts the mitochondrial respiratory chain by inhibiting complex I activities [5] and decreasing the mitochondrial membrane potential (MMP), leading to the release of apoptosis-related factors including cytochrome C into the cytoplasm. Caspase-9 activation then induces mitochondrial-dependent apoptosis. A balance is normally maintained between apoptosis and autophagy that ensures cellular homeostasis [6], which is disrupted in PD by the increase in apoptosis. Therapeutic strategies that restore this balance may therefore be effective for PD treatment.

Available pharmacological treatments for PD mitigate the disease symptoms without providing a cure [7]. Various symptoms including motor dysfunction emerge in PD patients after around 5 years of treatment. Therefore, more effective pharmacological options are needed. Piperine (PIP), an extract of *Piperlongum L.* that is used in traditional Chinese medicine, has demonstrated anti-inflammatory and anti-oxidative properties [8–10]. We previously reported that the combination of PIP and piperlongumine (PLG)—another alkaloid with similar properties as PIP [11, 12]—could inhibit apoptosis by blocking the opening of the mitochondrial permeability transition pore (mPTP), thereby exerting neuroprotective effects in a rat model of PD. However, it is unclear whether PIP alone has comparable effects against rotenone-induced PD while the underlying mechanism remains to be clarified.

To address these issues, the present study investigated the therapeutic effects of PIP in mouse and cellular models of rotenone-induced PD. PIP increased cell viability and activated autophagy by stimulating protein phosphotase 2A (PP2A) activity. The results suggest that PIP may be an effective agent for PD treatment.

## RESULTS

### PIP attenuates motor deficits induced by rotenone

**Figure 1:**
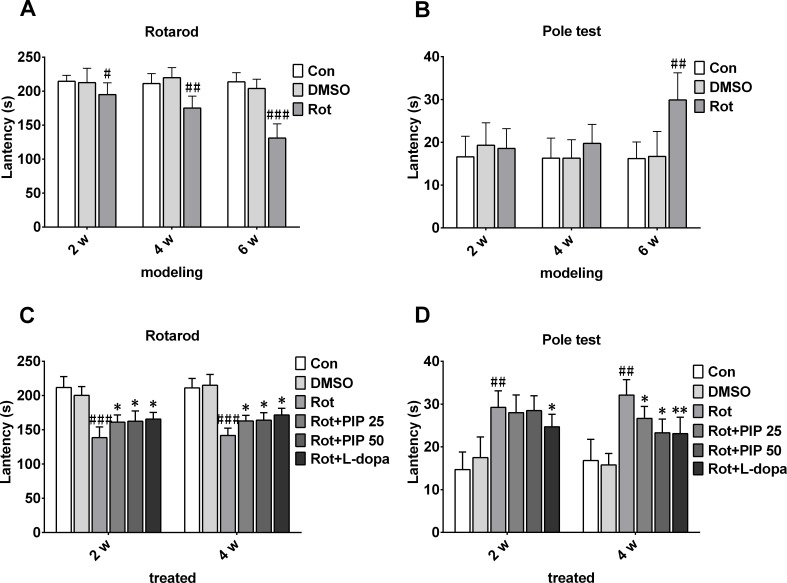
PIP reverses motor deficits induced by rotenone **A.**, **B.** C57BL mice were treated with rotenone (10mg/kg/day) for 6 weeks. Rotarod (A) and pole (B) tests were used to assess motor function. **C.**, **D.** Rotenone-treated mice were treated with PIP (25and 50mg/ml/day) or l-dopa (20mg/kg/day) for another 4 weeks, and then evaluated with rotarod (C) and pole (D) tests. Data are expressed as the mean ± SD (two-way analysis of variance). ^#^*P* < 0.05, ^##^*P* < 0.01, ^###^*P* < 0.001 *vs*. control (Con); *P < 0.05, **P < 0.01 *vs*. rotenone (Rot) (*n* = 10).

Motor dysfunction is a hallmark of PD [1]. To verify whether rotenone administration could induce motor deficits in mice, rotenone was orally administered at 10mg/kg for 6 weeks and motor behavior was measured by the rotarod and pole tests. Rotenone-treated animals were more likely to fall from the rod than control mice (Figure 1A) and took a longer time to descend the pole in the pole test, providing evidence for rotenone-induced locomotor dysfunction (Figure 1B).

To determine whether PIP could protect against the effects of rotenone, mice were treated with 25 or 50mg/kg PIP for 4 weeks after rotenone administration. PIP-treated animals remained on the Rotarod for a longer time than the rotenone-only group, though not as long as control animals. Mice treated with PIP as well as the positive control l-dopa descended the pole in the pole test in a shorter time than those in the rotenone group (Figure 1C, 1D).

### PIP mitigates the loss of TH-positive neurons in the substantia nigra(SN) and reduction in dopamine in the striatum induced by rotenone

**Figure 2:**
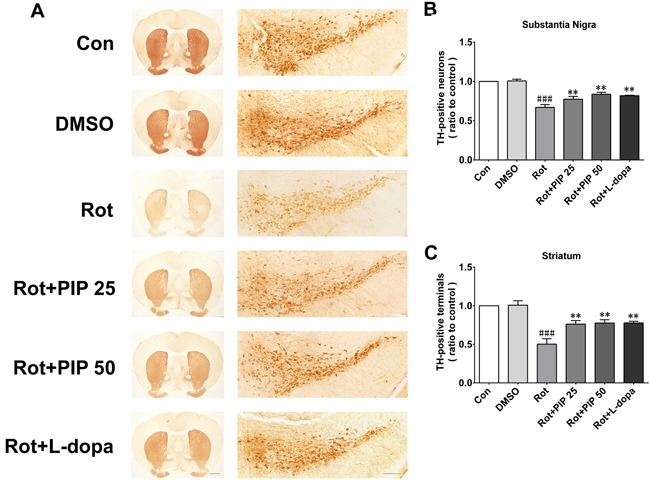
PIP reverses the reduction in TH-positive neurons induced by rotenone **A.** TH expression in substantia nigra (SN) and striatum sections were assessed by immunohistochemistry. Scale bar = 500 μM. **B.**, **C.** Quantitative analysis of TH-positive neurons in the SN (B) and striatum (C). Data are expressed as the mean ± SD (one-way analysis of variance). ^###^*P* < 0.001 *vs*. control (Con); **P < 0.01 *vs*. rotenone (Rot) (*n* = 5).

**Figure 3:**
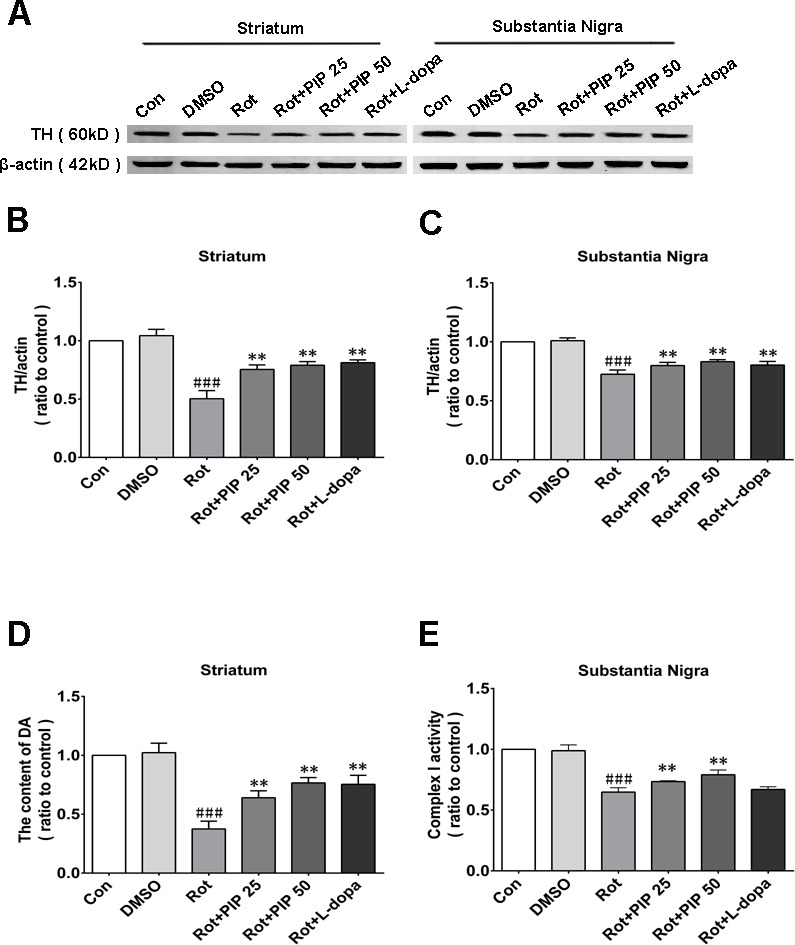
PIP restores TH expression reduced by rotenone **A.** TH expression in the SN and striatum, as determined by western blotting. **B.**, **C.** Quantitative analysis of TH expression in the SN (B) and striatum (C). **D.** Dopamine (DA) content in the striatum, as assessed by HPLC. **E.** Activity of mitochondrial complex I in the SN. Data are expressed as the mean ± SD (one-way analysis of variance) ^###^*P* < 0.001 *vs*. control (Con); **P < 0.01 *vs*. rotenone (Rot) (*n* = 5).

The nigrostriatal system plays a critical role in the control of motor output [13]. To determine whether this system is affected by rotenone treatment, sections of SN and striatum were assessed for the expression of TH, a dopaminergic neuron marker, by immunohistochemistry [14]. Rotenone treatment decreased the number of TH-positive neurons in both brain regions, an effect that was mitigated by PIP treatment (Figure 2). PIP was also found to reverse the decrease in TH protein level induced by rotenone by western blotting (Figure 3A-3C). Accordingly, an HPLC analysis showed that PIP blocked the rotenone-induced reduction in dopamine content in the striatum (Figure 3D), while it also rescued the activity of mitochondrial complex I (Figure 3E) that was suppressed by rotenone administration.

### PIP increases cell viability by enhancing mitochondrial function

**Figure 4:**
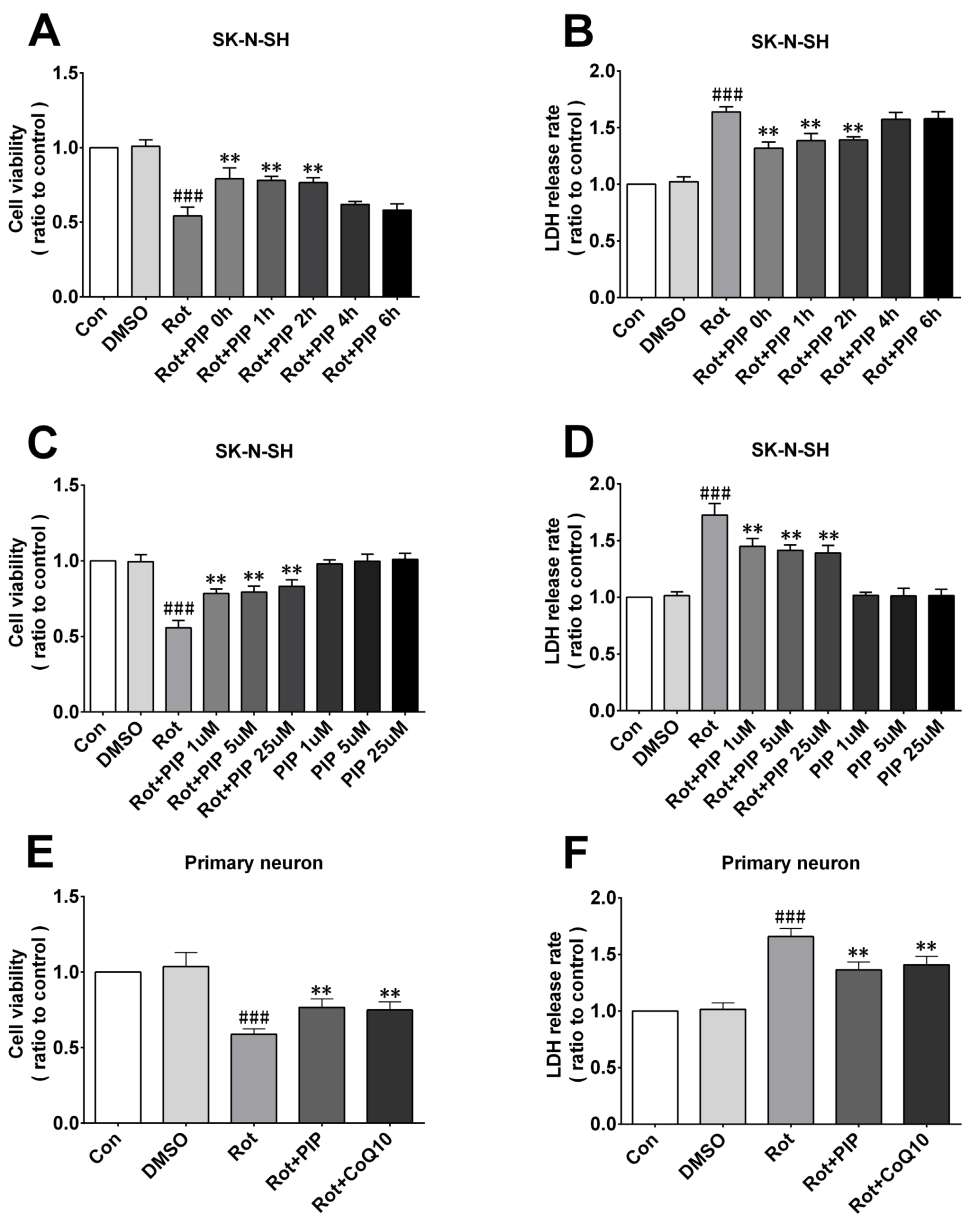
PIP enhances cell viability and reduces cytotoxicity induced by rotenone **A.**-**D.** Optimal PIP treatment time (A, B) and concentration (C, D) were determined in SK-N-SH cells by evaluating cell viability and cytotoxicity with the MTT and LDH assays, respectively. **E.**,**F.** Cell viability and cytotoxicity were detected in primary neurons with the MTT (E) and LDH (F) assays. Data are expressed as the mean ± SD (one-way analysis of variance) ^###^*P* < 0.001 *vs*. control (Con); **P < 0.01 *vs*. rotenone (Rot) (*n* = 3).

**Figure 5:**
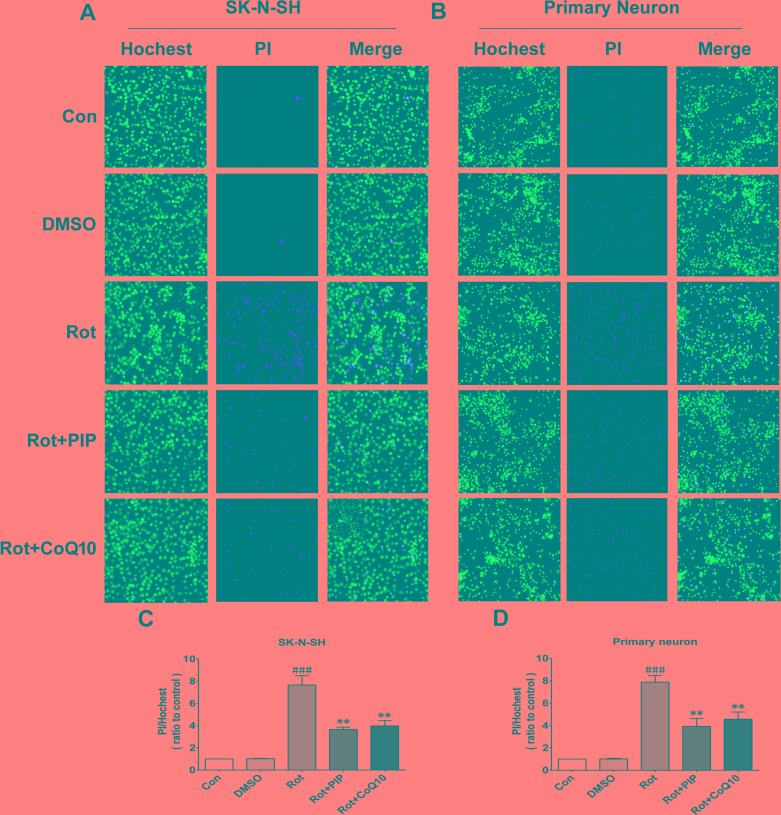
PIP reduces cell death rate in SK-N-SH cells and primary neurons treated with rotenone **A.**, **C.** Apoptotic SK-N-SH cells (A) and primary neurons (C) were identified by PI (red) and Hoechst (blue) staining. **B.**, **D.** Cell death rates were quantified in SK-N-SH cells (B) and primary neurons (D). Data are expressed as the mean ± SD (one-way analysis of variance). ^###^*P* < 0.001 *vs*. control (Con); **P < 0.01 *vs*. rotenone (Rot) (*n* = 3).

To investigate the mechanistic basis for the effects of PIP, the viability of SK-N-SH cells was assessed after treatment with a range of PIP concentrations (1, 5, and 25μM) and times (0, 1, 2, 4, and 6h after rotenone treatment). Rotenone (100nM) reduced cell viability, an effect that was reversed by PIP treatment in a dose- and time-dependent manner (Figure 4A, 4B), although this was not the case for 0and 1h of treatment (Figure 4C, 4D). Based on these results, cells were treated with 1μM PIP for 2h after rotenone treatment and co-treated for 24h in subsequent experiments. Similar results were obtained in primary neurons (Figure 4E, 4F). In addition, the rate of cell death was markedly suppressed by PIP treatment as compared to the rotenone-only group, as determined by PI/Hoechst staining (Figure 5).

**Figure 6:**
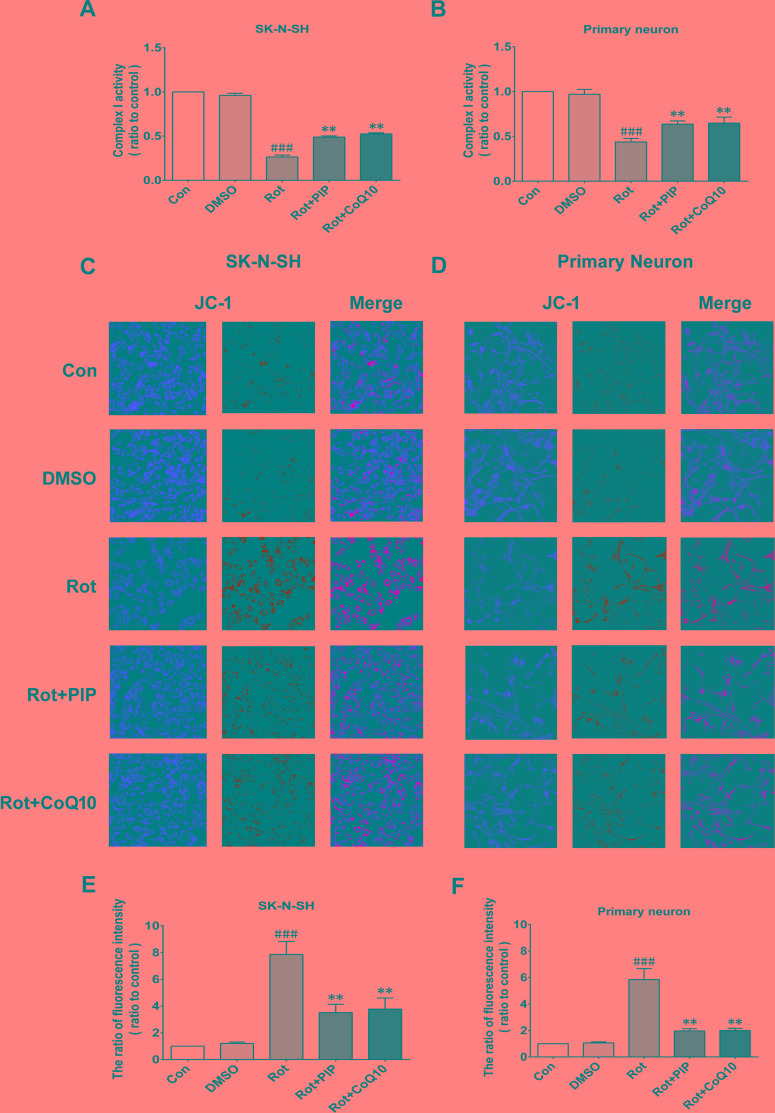
PIP reverses the decreases in mitochondrial complex I activity and MMP induced by rotenone **A.**, **B.** Mitochondrial complex I activity was detected in SK-N-SH cells (A) and primary neurons (B). **C.**, **D.** MMP was assessed by JC-1 staining in SK-N-SH cells (C) and primary neurons (D). **E.**, **F.** Quantitative analysis of fluorescence intensity. Data are expressed as the mean ± SD (one-way analysis of variance). ^###^*P* < 0.001 *vs*. control (Con); **P < 0.01 *vs*. rotenone (Rot) (*n* = 3).

**Figure 7:**
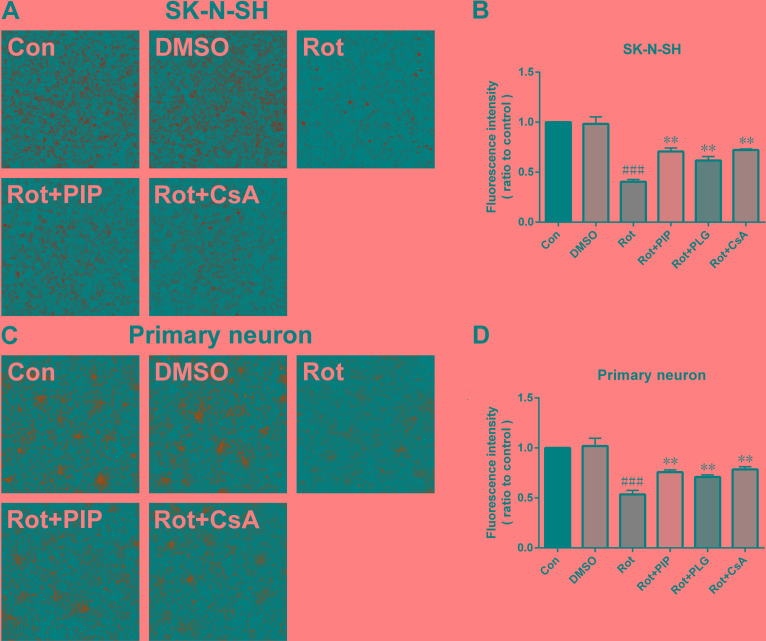
PIP blocks the opening of the mitochondrial permeability transition pore **A.**, **C.** Opening of mitochondrial permeability transition pore, as assessed by calcein-AM staining in SK-N-SH cells (A) and primary neurons (C). **B.**, **D.** Quantitative analysis of fluorescence intensity in SK-N-SH cells (B) and primary neurons (D). Data are expressed as the mean ± SD (one-way analysis of variance). ^###^*P* < 0.001 *vs*. control (Con); **P < 0.01 *vs*. rotenone (Rot) (*n* = 3).

Rotenone acts by inhibiting mitochondrial complex I. We therefore investigated whether PIP affects mitochondrial function. PIP treatment enhanced mitochondrial complex I activity in SK-N-SH cells and primary neurons, which was similar to the effect of coenzyme Q10 (Figure 6A, 6B). We also found using the JC-1 probe that PIP reversed the reduction in MMP caused by rotenone treatment (Figure 6C-6F). The opening of the mPTP induced by rotenone was also blocked in the presence of PIP, as determined using calcein-AM. Similar observations were made using cyclosporine A (Figure 7), an mPTP inhibitor [15]. Thus, PIP exerts therapeutic effects in SK-N-SH cells and primary neurons by enhancing mitochondrial function.

### PIP stimulates autophagy by suppressing mammalian target of rapamycin complex (mTORC) 1

**Figure 8:**
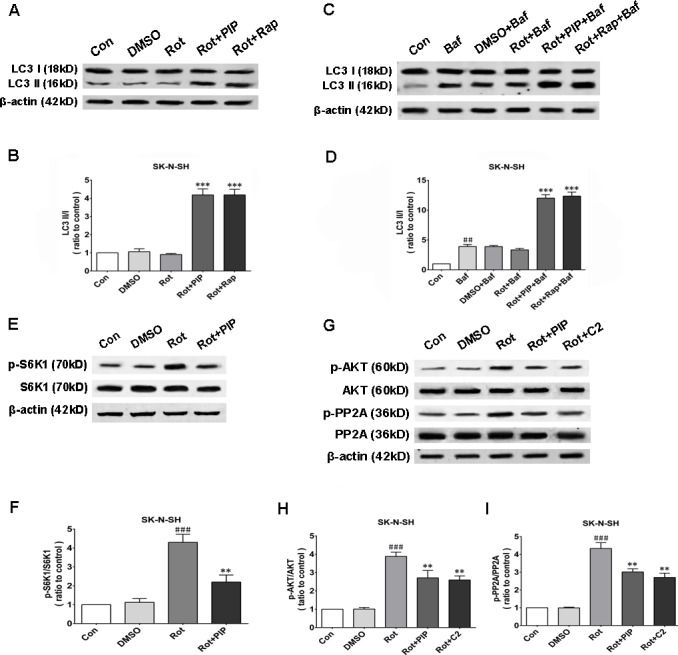
PIP induces autophagy *via* PP2A-dependent inhibition of AKT/mTOR signaling in SK-N-SH cells **A.**, **B.** Western blot analysis of LC3 I and LC3 II expression as a measure of autophagy induction (A) and quantification of LC3II/I ratio (B). **C.**, **D.** LC3 I and LC3 II expression in the presence of bafilomycin (Baf) (C) and quantification of LC3II/I ratio (D). **E.**, **F.** S6K1 and p-S6K1 expression as a measure of mTOR activity (E) and quantification of p-S6K1/S6K1 ratio (F). **G.**-**I.** Analysis of AKT, p-AKT, PP2A, and p-PP2A expression (G) and quantification of p-AKT/AKT (H) and p-PP2A/PP2A (I) ratio. Data are expressed as the mean ± SD (one-way analysis of variance). ^###^*P* < 0.001 *vs*. control (Con); **P < 0.01 *vs*. rotenone (Rot); ***P < 0.001 *vs*. rotenone (*n* = 3). Rap: rapamycin, C2: C2 ceramide.

**Figure 9:**
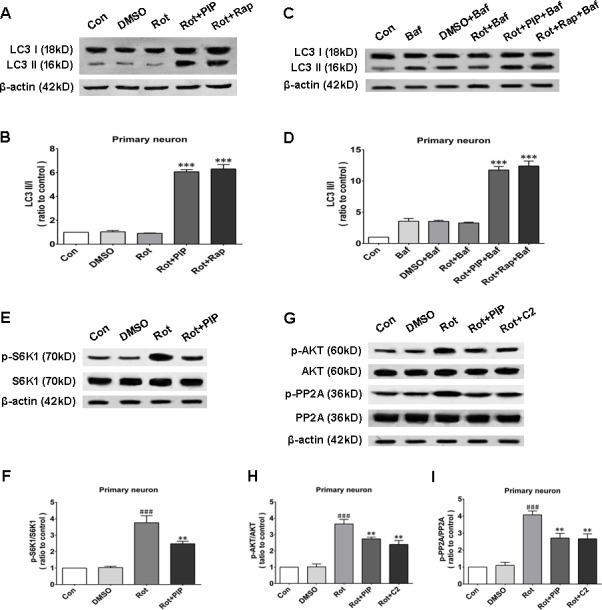
PIP induces autophagy *via* inhibition of PP2A-dependent AKT/mTOR signaling in primary neurons **A.**, **B.** Western blot analysis of LC3 I and LC3 II expression as a measure of autophagy induction (A) and quantification of LC3II/I ratio (B). **C.**, **D.** LC3 I and LC3 II expression in the presence of bafilomycin (Baf) (C) and quantification of LC3II/I ratio (D). **E.**, **F.** S6K1 and p-S6K1 expressionas a measure of mTOR activity (E) and quantification of p-S6K1/S6K1 ratio (F). **G.**-**I.** Analysis of AKT, p-AKT, PP2A, and p-PP2A expression (G) and quantification of p-AKT/AKT (H) and p-PP2A/PP2A (I) ratio. Data are expressed as the mean ± SD (one-way analysis of variance). ^###^*P* < 0.001 *vs*. control (Con); **P < 0.01 *vs*. rotenone(Rot); ***P < 0.001 *vs*. rotenone (*n* = 3). Rap: rapamycin, C2: C2 ceramide.

Autophagy is an important process for eliminating damaged mitochondria [16–18]. We therefore investigated whether PIP exerted protective effects against rotenone-induced injury by stimulating autophagy. The protein level of the autophagosomal marker LC3 II/I was increased by PIP or rapamycin treatment as compared to the rotenone and control groups, indicating that autophagy was activated in these mice (Figures 8A, 8B and 9A, 9B). Bafilomycin A1 inhibits the final steps of autophagy by blocking autophagosome-lysosome fusion; treatment with 100nM BafA1 for 6h increased LC3-II levels in the PIP and rapamycin groups relative to controls (Figures 8C, 8D and 9C, 9D). Unc-51-like autophagy-activating kinase (ULK) 1 is critical for triggering autophagy [19]; this is inhibited by its interaction with mTORC1 [20]. Suppression of mTORC1 results in the dissociation and activation of ULK1 and induction of autophagy. To determine whether this occurred upon PIP treatment, we assessed the activation of the mTOR1 substrate S6K. Rotenone treatment increased p-S6K level, implying activation of the upstream factor mTORC1 (Figures 8E, 8F and 9E, 9F). However, PIP treatment caused the downregulation of p-S6K level *via* suppression of mTORC1, leading to autophagy induction.

### PIP inhibits AKT *via* PP2A activation

**Figure 10:**
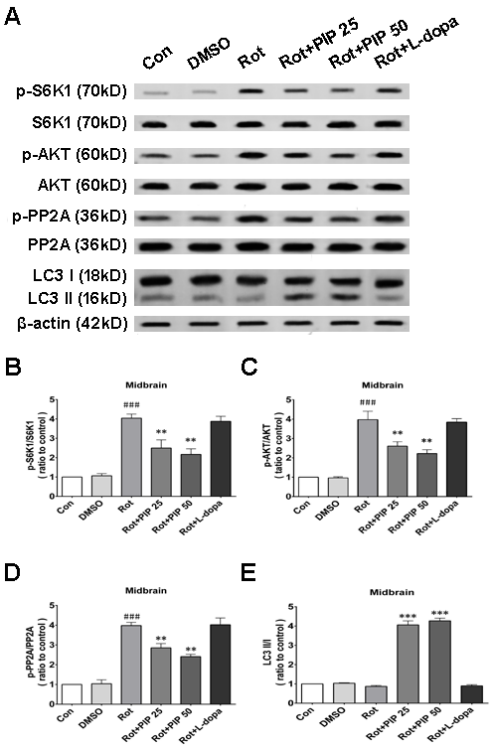
PIP induces autophagy *via* PP2Aactivation in a mouse model of rotenone-induced PD **A.** S6K, p-S6K1, AKT, p-AKT, PP2A, p-PP2A, LC3 I and LC3 II expression was determined by western blotting of SN tissue lysates; β-actin was used as a loading control. **B.**-**E.** Quantification of the ratios of p-S6K1/S6K1 (B), p-AKT/AKT (C), p-PP2A/PP2A (D), and LC3 II /I (E). Data are expressed as the mean ± SD (one-way analysis of variance). ^###^*P* < 0.001 *vs*. control (Con); *P < 0.05, **P < 0.01 *vs*. rotenone (Rot) (*n* = 3).

We next investigated whether factors upstream of mTORC1 are modulated by PIP treatment. mTOR interacts with and is necessary for the phosphorylation of AKT; the Akt/mTOR signaling pathway plays an important role in the modulation of autophagy [21]. Rotenone treatment increased AKT phosphorylation, an effect that was reversed by PIP treatment (Figures 8G, 8H and 9G, 9H). PP2A inactivates AKT *via* dephosphorylation at Ser473 [22]. Rotenone inactivates PP2A *via* Tyr307 phosphorylation [23]. The increase in PP2A Tyr307 phosphorylation was abolished in the presence of PIP or its agonist C2 ceramide (Figures 8G, 8I and 9G, 9I). These results indicate that rotenone suppressed PP2A activity *via* Tyr307 phosphorylation, which inhibited autophagy *via* AKT/mTOR signaling. However, p-PP2A (Tyr307) and p-AKT (Ser473) levels were restored by PIP or C2 ceramide treatment. Experiments in mice also confirmed that PIP but not l-dopa treatment induced autophagy by enhancing PP2A activity (Figure 10).

### Okadaic acid (OKA) blocks the induction of autophagy and the increase in cell viability resulting from PIP treatment *via* PP2A inactivation

**Figure 11:**
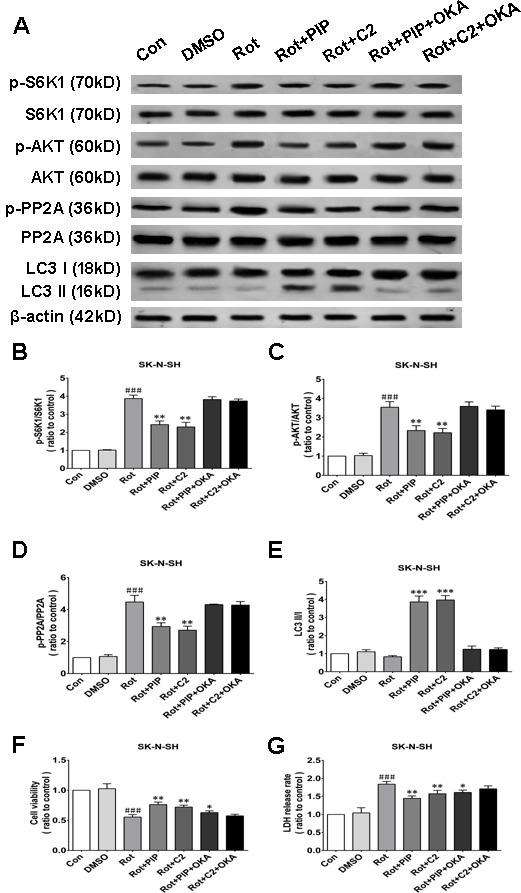
OKA reverses PIP-induced autophagy and increase in cell viability *via* PP2A inactivation in SK-N-SH cells **A.** S6K, p-S6K1, AKT, p-AKT, PP2A, p-PP2A, LC3 I, and LC3 II expression was determined by western blotting following OKA treatment; β-actin was used as a loading control.**B.**-**E.** Quantification of the ratios of p-S6K1/S6K1 (B), p-AKT/AKT (C), p-PP2A/PP2A (D), and LC3 II /I (E). **F.**, **G.** Detection of cell viability (F) and cytotoxicity (G) with the MTT and LDH assays, respectively. Data are expressed as the mean ± SD (one-way analysis of variance). ^###^*P* < 0.001 *vs*. control (Con); *P < 0.05, **P < 0.01 *vs*. rotenone (Rot) (*n* = 3). C2: C2 ceramide, OKA: okadaic acid.

**Figure 12:**
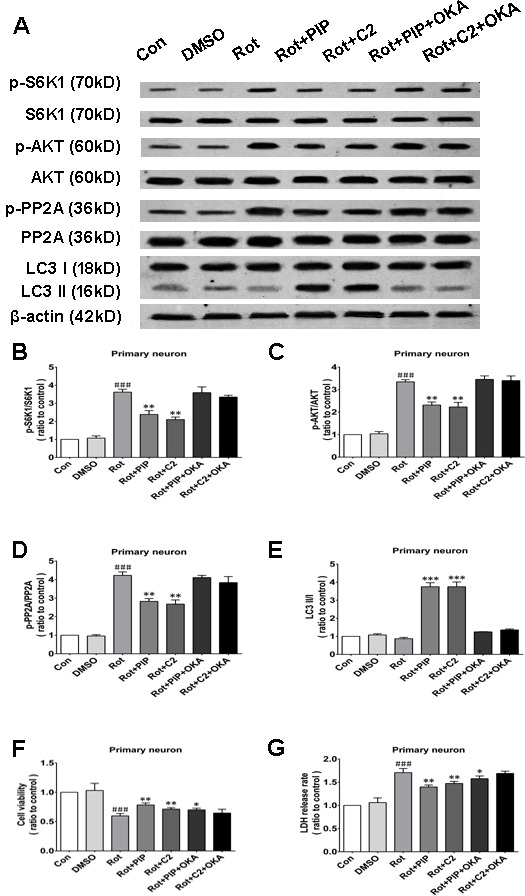
OKA reverses PIP-induced autophagy and increase in cell viability *via* PP2A inactivation in primary neurons **A.** S6K, p-S6K1, AKT, p-AKT, PP2A, p-PP2A, LC3 I, and LC3 II expression was determined by western blotting following OKA treatment; β-actin was used as a loading control.**B.**-**E.** Quantification of the ratios of p-S6K1/S6K1 (B), p-AKT/AKT (C), p-PP2A/PP2A (D), and LC3 II /I (E). **F.**, **G.** Detection of cell viability (F) and cytotoxicity (G) with the MTT and LDH assays, respectively. Data are expressed as the mean ± SD (one-way analysis of variance). ^###^*P* < 0.001 *vs*. control (Con); *P < 0.05, **P < 0.01 *vs*. rotenone (Rot) (*n* = 3). C2: C2 ceramide, OKA: okadaic acid.

OKA, a pharmacological inhibitor of PP2A [24], suppressed PP2A activity regardless of PIP or C2ceramide treatment. In addition, there was no variation in LC3-II levels between rotenone and PIP/OKA groups, indicating that OKA suppressed PIP-induced autophagy (Figures 11A-11E and 12A-12E). To determine whether PIP still exerts protection in the absence of PP2A activity, we assessed viability in cells treated with OKA. Combined treatment with PIP/OKA slightly improved cell viability, but the difference between the rotenone and PIP/OKA groups was not statistically significant (Figures 11F, 11G and 12F, 12G). These results indicate that PP2A activity is necessary for the therapeutic effect of PIP.

### PIP suppresses apoptosis induced by rotenone

**Figure 13:**
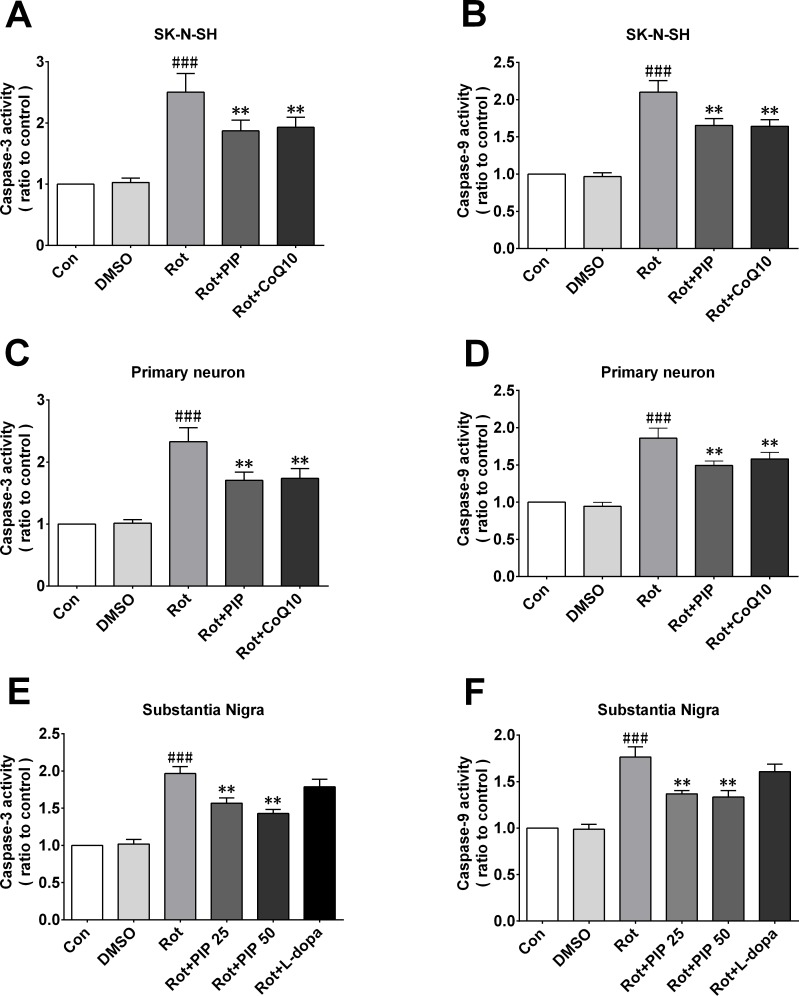
PIP inhibits rotenone-induced apoptosis **A.**, **C.**, **E.** Caspase-3 expressionin SK-N-SH cells (A), primary neurons (C), and the SN of rotenone-treated mice (E). **B.**, **D.**, **F.** Caspase-9 expression in SK-N-SH cells (B), primary neurons (D), and the SN of rotenone-treated mice. Data are expressed as the mean ± SD (one-way analysis of variance). ^###^*P* < 0.001 *vs*. control (Con); **P < 0.01 *vs*. rotenone (Rot) (*n* = 3).

To determine whether PIP-induced autophagy could suppress apoptosis by eliminating mitochondria damaged by rotenone, we measured caspase-3 and -9 activities. The former activates both intrinsic and extrinsic apoptotic pathways, while the latter stimulates the intrinsic or mitochondrial apoptotic pathway [25]. Caspase-3 and -9 were activated by rotenone treatment in SK-N-SH cells and primary neurons; however, this increase in activity was inhibited by PIP treatment (Figure 13A-13D). Similar observations were made in mice (Figure 13E, 13F).

## DISCUSSION

**Figure 14:**
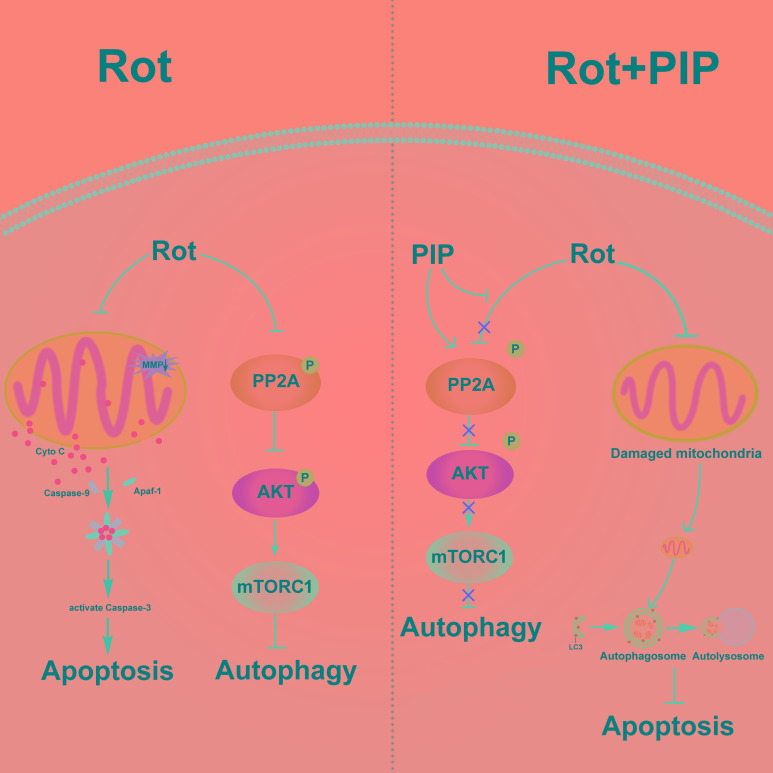
PIP exerts neuroprotective effects against rotenone-induced injury by activating PP2A and restoring the balance between autophagy and apoptosis Rotenone injures mitochondria by disrupting MMP, leading to the release cytochrome (Cyto) C from mitochondria into the cytosol followed by induction of apoptosis. Meanwhile, rotenone inhibits autophagy by suppressing PP2A activity. PIP restores PP2A activity and induces autophagy, which clears injured mitochondria and suppresses apoptosis. MMP: mitochondrial membrane potential; PIP: piperine; PP2A: protein phosphotase 2A.

The current study confirmed that PIP has a therapeutic effect in rotenone-induced PD models that is exerted *via* activation of PP2A and induction of autophagy (Figure 14).

There are currently few therapeutic options for the treatment of PD. One of these is l-dopa, a dopamine supplement that has many disadvantages including a short half-life and variable effectiveness in improving motor symptoms [26]. In this study, we investigated PIP as a potential agent for PD treatment using cellular and mouse models of PD induced by rotenone. PIP administration improved motor performance on the rotarod and pole tests in rotenone-treated mice and mitigated the loss of dopaminergic neurons and the decrease in dopamine levels in the SN and striatum, respectively. It also protected SK-N-SH cells and primary neurons against mitochondrial damage resulting from rotenone treatment by activating autophagy *via* inhibition of AKT-mTORC1 signaling, with PP2A mediating this effect.

PIP has previously been reported to have neuroprotective effects in a variety of PD models, including those induced by 1-methyl-4-phenyl-1, 2, 3, 6-tetrahydropyridine (MPTP) [27–29], rotenone [30], and 6-hydroxydopamine (6-OHDA) [31]. PIP (10mg/kg) administration for 15 days—including 8 days of pretreatment—attenuated MPTP-induced motor deficits and cognitive dysfunction by enhancing resistance to apoptosis *via* maintenance of the balance between B cell lymphoma (Bcl)-2 and Bcl-2-associated X protein [27]. Our previous study showed that combined treatment with PIP and PLG had protective effects in rotenone-induced PD models, including blocking mPTP opening and apoptosis [30]. PIP pretreatment exerted a protective effect in a 6-OHDA-induced PD model *via* anti-apoptotic and anti-inflammatory mechanisms [31], and may also be effective for the treatment of AD [32–34], depression [35, 36], and other types of cognitive dysfunction [37, 38]. The present study investigated the therapeutic and not the preventive effects of PIP, and found that these involve anti-apoptotic as well as autophagy-promoting mechanisms.

The balance between autophagy and apoptosis is critical for maintaining cellular homeostasis [39]. This balance is perturbed in various disorders, including neurodegenerative diseases. An increase in mitochondrial-dependent apoptosis has been detected in PD [40–42], and impairment of autophagy has been reported in the brains of PD patients [43–45]. Treatment strategies that restore the balance of apoptosis and autophagy have been proposed for PD [6, 46], and many agents have been discovered or developed that target autophagy activation. For example, sestrin2 activated autophagy and suppressed rotenone-induced cytotoxicity by upregulating AMP-activated protein *kinase* activity and exerted a protective effect on dopaminergic cells [47]. Conophylline, a vinca alkaloid, protected against MPTP-induced cell death by activating mTOR-independent autophagy [48]. Erythropoietin provided protection against rotenone-induced neurotoxicity, which involved restoration of Beclin-1 expression and downregulation of mTOR [49]. In the present study, PIP stimulated PP2A activity by dephosphorylation at Tyr307; autophagy was induced *via* suppression of AKT/mTORC1 signaling by PP2A-mediated AKT dephosphorylation, which mitigated damage to mitochondria and inhibited apoptosis by blocking the release of pro-apoptotic factors into the cytosol.

In conclusion, our results provide evidence that PIP exerts therapeutic effects by restoring the balance between apoptosis and autophagy, and suggest that PIP can potentially be effective in the treatment of PD.

## MATERIALS AND METHODS

### Animals

C57BL male mice, 3 months old, were purchased from Vital River Laboratories (Beijing, China) and housed at room temperature (22°C-25°C) under a12:12-h light/dark cycle. Animal experiments conformed to the National Institutes of Health (NIH; Bethesda, MD, USA) guidelines for animal care and use. Mice were randomly divided into six groups. Control group and dimethyl sulfoxide (DMSO) groups received normal saline and DMSO, respectively; the rotenone groups were orally administered rotenone (10mg/kg) for 6 weeks, after which they received PIP (25 mg/kg) or 3, 4-dihydroxyphenyl-l-alanine(l-dopa; 50 mg/kg) by oral administration for 4 weeks.

### Reagents

Antibodies against the following proteins were used in the study: tyrosine hydroxylase (TH) and β-actin (both 1:2000; from Sigma, St. Louis, MO, USA); PP2A (1:1,000; BD Biosciences, Franklin Lakes, NJ, USA); phospho- (p-)PP2A (Tyr307) (1:1000; Abcam, Cambridge, UK); microtubule-associated protein 1A/1Blight chain (LC)3 (1:1,000; Novus Biologicals, Littleton, CO, USA); AKT, p-AKT, and ribosomal protein S6 kinase (S6K) (all from Cell Signaling Technology, Danvers, MA, USA); and p-S6K (Millipore, Billerica, MA, USA). Fluorophore-conjugated secondary antibodies (Invitrogen, Carlsbad, CA, USA) were used at 1:10,000 dilutions. Rotenone (Sigma) and PIP were obtained as previously described [50]; l-dopa was purchased from Sigma.

### Rotarod test

The rotarod test was used to evaluate motor functioning [51]. Mice were trained once a day for 3days with rotation speeds of 10, 12, and 15 rpm on days 1-3. The time that the mouse remained on the rod when tested on the three occasions was recorded and averaged.

### Pole test

The pole test was used to evaluate the degree of bradykinesia and was carried out as previously described [52]. Briefly, a mouse was placed at the top of a pole with a length of 50cmand radius of 4mm radius, and the time taken for the mouse to reach the ground was recorded over three trials and averaged.

### Immunohistochemistry

Immunohistochemistry was carried out as previously described [53]. Mice were anesthetized with 8% chloral hydrate and perfused with physiological saline followed by 4% paraformaldehyde. Then, the brain was removed and dehydrated in 20% and 30% sucrose solution, then sectioned at a thickness of 20μm. Sections were rinsed in phosphate-buffered saline (PBS) and then incubated in 3% H_2_O_2_ for 10min to block endogenous peroxidase activity. After washing in PBS, sections were incubated in 10% goat serum followed by 0.1% TritonX-100 in PBS for 60min, then incubated overnight at 4°C in anti-TH antibody. A biotinylated goat anti-mouse secondary antibody (1:200; Zhongshan Golden Bridge Biotechnology Co., Beijing, China) and diaminobenzidine were used to detect immunoreactivity. 20 successive sections were selected from each brain to undergo examination. Unbiased stereology was used to estimate the number of dopaminergic neurons of each section, which proformed under the 40x objective of Leica DM5000B microscope (Leica Microsystems, Bannockburn, IL) and analyzed with Stereo Investigator software (MBF Bioscience, Williston, VT). The total TH-positive neurons were generated through the optical fractionator probe. 5 mice from each group were used in this measurement. The Coefficient of Error (Gundersen) for the individual counts was 0.05 routinely.

### Measurement of dopamine levels by high-performance liquid chromatography (HPLC)

Striatum tissue was weighed and homogenized in 0.1mol/l HClO_4_ and incubated on ice for 1h, then centrifuged at 12,000rpm at 4°C for 20 min. The supernatant was mixed with the HPLC mobile phase consisting of 63.5mM citric acid monohydrate, 60.9mM trisodium citrate dehydrate, 0.1M EDTA, and 0.5M C-8alkyl sulfonate (pH 4.3) and injected into the HPLC column under analytical conditions. Data are expressed as pg dopamine per mg tissue.

### Determination of mitochondrial complex I activity

Mitochondrial complex I activity was evaluated using the Mitochondrial Complex I Activity Assay kit (AAMT001-1KIT; Novagen, Madison, WI, USA) according to the manufacturer's protocol. The brains of mice quickly removed after killed. The striatum was dissected out. The midbrain was divided into two parts from the ventral side perpendicular to the long axis of mesencephalon with a cut exactly. The tail buds were eminent at the caudal border. The substantiae nigra in two were then easily identified and freely dissected. Cells and tissue were frozen and thawed three times as previously described [54] and protein concentration was measured with the Bicinchoninic Acid Protein Assay kit (23225; Pierce, Rockford, IL, USA). Cell and tissue lysates were incubated with complex I capture antibody for 3 h at room temperature, and complex I activity was determined by measuring the oxidation of nicotinamide adenine dinucleotide (NAD)H to NAD+, which was observed as a reduction in the dye and a corresponding increase in absorbance at 450 nm after 30 min. Each sample was tested in duplicate and activity is expressed as the change in absorbance per minute per amount of sample loaded in the well. Two readings related to sample activity can be generated, respectively are total protein activity and complex I specific activity. The ratios of specific activity to total activity in each group were used to analysis.

### Measurement of caspase-3 activity

Cell or tissue samples (5 mg) were homogenized and incubated in 120 μl lysis buffer on ice for 10 min, then centrifuged at 12,000 rpm for 10 min at 4°C. The soluble fraction was transferred to a 1.5-ml tube, and protein concentration was determined with a Bradford assay kit (GenMed Scientifics, Shanghai, China). Caspase-3 activity was assessed using the Caspase-3 Colorimetric Assay kit (Applygen Technologies, Beijing, China) according to the manufacturer's instructions.

### Measurement of caspase-9 activity

Caspase-9 activity was determined using the Caspase-9 Colorimetric Assay kit (C1119; Applygen Technologies, Beijing, China) based on spectro photometric detection of the chromophore p-nitroanilide (pNA) after its cleavage from the labeled substrate LEHD-pNA. A 60-μl volume of cell lysis buffer was added to the cell pellet, which was vortexed and incubated on ice for 15 min, followed by centrifugation at 12,000 × *g* for 10 min at 4°C. Protein concentration was determined using a Bradford assay kit (500-0006; Bio-Rad, Hercules, CA, USA), and 50 μl protein were added to a 96-well plate along with 45 μl reaction buffer and 5 μl of 2 mM LEHD-pNA (for a final concentration of 100 μM). After incubation at 37°C for 2 h, absorbance was read at 405 nm on a spectrophotometer. One unit of activity was defined as the amount of enzyme that cleaved 1.0 nmol of the pNA substrate per hour at 37°C under saturated substrate concentrations.

### Cell viability and lactate dehydrogenase (LDH) assays

Cell viability was determined with the 3-(4,5-dimethylthiazol-2-yl)-2,5-diphenyltetrazolium bromide (MTT) assay as previously described. Briefly, cells were seeded in a 96-well microplate (1×10^4^ cells/well) and cultured for 24h. The medium was replaced by MTT (Promega, Madison, WI, USA) at a final concentration of 0.5mg/ml, followed by incubation for 4h. Cells were washed twice with PBS and formazan crystals were dissolved in 100 μl DMSO. Absorbance was read at 490nm with a microplate reader (PerkinElmer, Waltham, MA, USA).

The LDH assay was carried out using a cyto toxicity detection kit (Roche Diagnostics, Mannheim, Germany) according to a previously published protocol. LDH release was measured in a 100-μl aliquot of supernatant, with 100μl preservation solution used as a blank to correct the optical density reading at 490nm. Each sample was tested in triplicate, and the half-maximal effective concentration was averaged from five experiments.

### Propidium iodide (PI)/Hoechst staining

Apoptotic cells were identified by labeling with PI (4 μM) and Hoechst 33432 (0.5 μg/ml) (both from Sigma) for 10 min at 37°C. PI-positive cells were counted under an epifluorescence microscope at excitation and emission wavelengths of 535 and 615nm, respectively. Images were acquired by high content analysis to evaluate changes in PI signal.

### JC-1 staining

The MMP of cells was measured using JC-1 (T4069; Sigma), a dual-emission membrane potential-sensitive probe that exists as a green fluorescent monomer at a low MMP and forms aggregates withred/orange fluorescence at a higher MMP. JC-1 (1.3 μg/ml). Cells cultured in 24-well plates were washed twice with PBS and the probe was added for 30 min at 37°C. The change in fluorescence at 488/530 nm (green) and 549/595 nm (red) was monitored by high-content screening, and the ratio of green to red fluorescence intensity was determined.

### Evaluation of mPTP activation with calcein-AM

Cells were grown in a 24-well plate and mPTP activation was determined by monitoring calcein-AM fluorescence using the MitoProbe Transition Pore Assay kit (M34153; Invitrogen) according to the recommended protocol. Briefly, cells were incubated with calcein-AM and CoCl_2_ with or without ionomycin in Hank's Balanced Salt Solution (HBSS)/Ca^2+^ at 37°C for 15 min while protected from light. After two washes with HBSS/Ca^2+^, calcein-AM fluorescence was detected by high-content screening at 488/530 nm.

### Western blotting

Western blotting was carried out as previously described [55]. Cells or tissue were lysed on ice for 30 min in buffer containing1%Nonidet P-40 (Calbiochem, San Diego, CA, USA), 150 mM NaCl, 0.5% deoxycholicacid, 50 mM Tris-HCl(pH7.4), 0.1% sodium dodecyl sulfate (SDS), and protease and phosphatase inhibitors (Roche, Indianapolis, IN, USA). Cell extracts were centrifuged at 12,000 × *g* for 30 min at 4°C, and the supernatant was resolved by 12%-15% Bis-Tris SDS-polyacrylamide gel electrophoresis and transferred to polyvinylidene difluoride membranes that were probed overnight at 4°C for TH, p-PP2A, PP2A, p-AKT, AKT, p-S6K, S6K, LC3, and β-actin expression. Membranes were incubated with secondary antibody (LI-COR, Lincoln, NE, USA) and protein bands were visualized by enhanced chemiluminescence and quantified using ImageJ software (NIH).

### Primary neuronal culture

All experiments were authorized by the Institutional Animal Care and Use Committee of Capital Medical University of Science and Technology (approval No. SCXK-2011-004) and performed according to the NIH Guide for the Care and Use of Laboratory Animals. Surgeries were performed under chloral hydrate anesthesia. Primary neurons were prepared from brains of Sprague-Dawley rat embryos (day14.5-15.5). Dissociated neurons were cultured in 6- or 24-well plates on cover slips coated with 100 mg/ml poly-l-lysine (Sigma, P1524) in Neurobasal medium (Gibco, 21103-049) supplemented with l-glutamine (0.5 mM) and 50× B27 supplement (fora final concentration of 1×; Gibco, 17504-044). Primary neurons were treated with rotenone after 7 days of culture.

### Statistical analysis

Western blots were analyzed as previously described [56]. Relative optical density values for bands corresponding to TH and LC3 were normalized to the value for β-actin, while p-PP2A, p-AKT, and p-S6K levels were normalized to those of PP2A, AKT, and S6K, respectively. The normalized ratio for the control group was taken as 1. For caspase-3 and -9 activity, cell viability, and LDH assays, values for the control group were taken as 1. Statistical analysis was carried outusing Prism 5 (GraphPad Software, La Jolla, CA, USA). Results are presented as mean ± SD of at least three independent experiments. Differences between groups were evaluated by analysis of variance. Differences were considered significant at *P* < 0.05.

## References

[1] Kalia LV, Lang AE (2015). Parkinson's disease. LANCET.

[2] Olanow CW, Stern MB (2008). Parkinson's disease: unresolved issues. ANN NEUROL.

[3] Cannon JR, Tapias V, Na HM, Honick AS, Drolet RE, Greenamyre JT (2009). A highly reproducible rotenone model of Parkinson's disease. NEUROBIOL DIS.

[4] Johnson ME, Bobrovskaya L (2015). An update on the rotenone models of Parkinson's disease: their ability to reproduce the features of clinical disease and model gene-environment interactions. NEUROTOXICOLOGY.

[5] Hirst J (2013). Mitochondrial complex I. ANNU REV BIOCHEM.

[6] Ghavami S, Shojaei S, Yeganeh B, Ande SR, Jangamreddy JR, Mehrpour M, Christoffersson J, Chaabane W, Moghadam AR, Kashani HH, Hashemi M, Owji AA, Los MJ (2014). Autophagy and apoptosis dysfunction in neurodegenerative disorders. PROG NEUROBIOL.

[7] Lees AJ, Hardy J, Revesz T (2009). Parkinson's disease. LANCET.

[8] Lu Y, Liu J, Li H, Gu L (2016). Piperine Ameliorates Lipopolysaccharide-Induced Acute Lung Injury via Modulating NF-kappaB Signaling Pathways. INFLAMMATION.

[9] Kumar S, Kamboj J, Suman, Sharma S (2011). Overview for various aspects of the health benefits of Piper longum linn. fruit. J Acupunct Meridian Stud.

[10] Krishna MS, Joy B, Sundaresan A (2015). Effect on oxidative stress, glucose uptake level and lipid droplet content by Apigenin 7, 4′-dimethyl ether isolated from Piper longum L. J Food Sci Technol.

[11] Lee W, Yoo H, Kim JA, Lee S, Jee JG, Lee MY, Lee YM, Bae JS (2013). Barrier protective effects of piperlonguminine in LPS-induced inflammation in vitro and in vivo. FOOD CHEM TOXICOL.

[12] Raj L, Ide T, Gurkar AU, Foley M, Schenone M, Li X, Tolliday NJ, Golub TR, Carr SA, Shamji AF, Stern AM, Mandinova A, Schreiber SL, Lee SW (2011). Selective killing of cancer cells by a small molecule targeting the stress response to ROS. NATURE.

[13] Samii A, Nutt JG, Ransom BR (2004). Parkinson's disease. LANCET.

[14] Calvo AC, Pey AL, Miranda-Vizuete A, Doskeland AP, Martinez A (2011). Divergence in enzyme regulation between Caenorhabditis elegans and human tyrosine hydroxylase, the key enzyme in the synthesis of dopamine. BIOCHEM J.

[15] Fernandes MP, Leite AC, Araujo FF, Saad ST, Baratti MO, Correia MT, Coelho LC, Gadelha FR, Vercesi AE (2014). The Cratylia mollis seed lectin induces membrane permeability transition in isolated rat liver mitochondria and a cyclosporine a-insensitive permeability transition in Trypanosoma cruzi mitochondria. J EUKARYOT MICROBIOL.

[16] Luo Y, Hoffer A, Hoffer B, Qi X (2015). Mitochondria: A Therapeutic Target for Parkinson's Disease?. INT J MOL SCI.

[17] Wang K, Klionsky DJ (2011). Mitochondria removal by autophagy. AUTOPHAGY.

[18] Narendra DP, Youle RJ (2011). Targeting mitochondrial dysfunction: role for PINK1 and Parkin in mitochondrial quality control. Antioxid Redox Signal.

[19] Mizushima N (2010). The role of the Atg1/ULK1 complex in autophagy regulation. CURR OPIN CELL BIOL.

[20] Jung CH, Ro SH, Cao J, Otto NM, Kim DH (2010). mTOR regulation of autophagy. FEBS LETT.

[21] Heras-Sandoval D, Perez-Rojas JM, Hernandez-Damian J, Pedraza-Chaverri J (2014). The role of PI3K/AKT/mTOR pathway in the modulation of autophagy and the clearance of protein aggregates in neurodegeneration. CELL SIGNAL.

[22] Dennis MD, Coleman CS, Berg A, Jefferson LS, Kimball SR (2014). REDD1 enhances protein phosphatase 2A-mediated dephosphorylation of Akt to repress mTORC1 signaling. SCI SIGNAL.

[23] Chen J, Martin BL, Brautigan DL (1992). Regulation of protein serine-threonine phosphatase type-2A by tyrosine phosphorylation. SCIENCE.

[24] Hata Y, Timalsina S, Maimaiti S (2013). Okadaic Acid: a tool to study the hippo pathway. MAR DRUGS.

[25] Kim B, Srivastava SK, Kim SH (2015). Caspase-9 as a therapeutic target for treating cancer. Expert Opin Ther Targets.

[26] Connolly BS, Lang AE (2014). Pharmacological treatment of Parkinson disease: a review. JAMA.

[27] Yang W, Chen YH, Liu H, Qu HD (2015). Neuroprotective effects of piperine on the 1-methyl-4-phenyl-1,2,3,6-tetrahydropyridine-induced Parkinson's disease mouse model. INT J MOL MED.

[28] Bi Y, Qu PC, Wang QS, Zheng L, Liu HL, Luo R, Chen XQ, Ba YY, Wu X, Yang H (2015). Neuroprotective effects of alkaloids from Piper longum in a MPTP-induced mouse model of Parkinson's disease. PHARM BIOL.

[29] Lee CS, Han ES, Kim YK (2006). Piperine inhibition of 1-methyl-4-phenylpyridinium-induced mitochondrial dysfunction and cell death in PC12 cells. EUR J PHARMACOL.

[30] Wang H, Liu J, Gao G, Wu X, Wang X, Yang H (2015). Protection effect of piperine and piperlonguminine from Piper longum L. alkaloids against rotenone-induced neuronal injury. BRAIN RES.

[31] Shrivastava P, Vaibhav K, Tabassum R, Khan A, Ishrat T, Khan MM, Ahmad A, Islam F, Safhi MM, Islam F (2013). Anti-apoptotic and anti-inflammatory effect of Piperine on 6-OHDA induced Parkinson's rat model. J NUTR BIOCHEM.

[32] Elnaggar YS, Etman SM, Abdelmonsif DA, Abdallah OY (2015). Intranasal Piperine-Loaded Chitosan Nanoparticles as Brain-Targeted Therapy in Alzheimer's Disease: Optimization, Biological Efficacy, and Potential Toxicity. J Pharm Sci.

[33] Chonpathompikunlert P, Wattanathorn J, Muchimapura S (2010). Piperine, the main alkaloid of Thai black pepper, protects against neurodegeneration and cognitive impairment in animal model of cognitive deficit like condition of Alzheimer's disease. FOOD CHEM TOXICOL.

[34] Chonpathompikunlert P, Yoshitomi T, Han J, Isoda H, Nagasaki Y (2011). The use of nitroxide radical-containing nanoparticles coupled with piperine to protect neuroblastoma SH-SY5Y cells from Abeta-induced oxidative stress. BIOMATERIALS.

[35] Borre YE, Panagaki T, Koelink PJ, Morgan ME, Hendriksen H, Garssen J, Kraneveld AD, Olivier B, Oosting RS (2014). Neuroprotective and cognitive enhancing effects of a multi-targeted food intervention in an animal model of neurodegeneration and depression. NEUROPHARMACOLOGY.

[36] Rinwa P, Kumar A, Garg S (2013). Suppression of neuroinflammatory and apoptotic signaling cascade by curcumin alone and in combination with piperine in rat model of olfactory bulbectomy induced depression. PLOS ONE.

[37] Rinwa P, Kumar A (2013). Quercetin along with piperine prevents cognitive dysfunction, oxidative stress and neuro-inflammation associated with mouse model of chronic unpredictable stress. ARCH PHARM RES.

[38] Rinwa P, Kumar A (2012). Piperine potentiates the protective effects of curcumin against chronic unpredictable stress-induced cognitive impairment and oxidative damage in mice. BRAIN RES.

[39] Eisenberg-Lerner A, Bialik S, Simon HU, Kimchi A (2009). Life and death partners: apoptosis, autophagy and the cross-talk between them. CELL DEATH DIFFER.

[40] Perier C, Bove J, Vila M (2012). Mitochondria and programmed cell death in Parkinson's disease: apoptosis and beyond. Antioxid Redox Signal.

[41] Perier C, Tieu K, Guegan C, Caspersen C, Jackson-Lewis V, Carelli V, Martinuzzi A, Hirano M, Przedborski S, Vila M (2005). Complex I deficiency primes Bax-dependent neuronal apoptosis through mitochondrial oxidative damage. Proc Natl Acad Sci U S A.

[42] Perier C, Bove J, Dehay B, Jackson-Lewis V, Rabinovitch PS, Przedborski S, Vila M (2010). Apoptosis-inducing factor deficiency sensitizes dopaminergic neurons to parkinsonian neurotoxins. ANN NEUROL.

[43] Xilouri M, Stefanis L (2011). Autophagic pathways in Parkinson disease and related disorders. EXPERT REV MOL MED.

[44] Pan T, Kondo S, Le W, Jankovic J (2008). The role of autophagy-lysosome pathway in neurodegeneration associated with Parkinson's disease. BRAIN.

[45] Banerjee R, Beal MF, Thomas B (2010). Autophagy in neurodegenerative disorders: pathogenic roles and therapeutic implications. TRENDS NEUROSCI.

[46] Franco-Iborra S, Vila M, Perier C (2015). The Parkinson Disease Mitochondrial Hypothesis: Where Are We at?. NEUROSCIENTIST.

[47] Hou YS, Guan JJ, Xu HD, Wu F, Sheng R, Qin ZH (2015). Sestrin2 Protects Dopaminergic Cells against Rotenone Toxicity through AMPK-Dependent Autophagy Activation. MOL CELL BIOL.

[48] Sasazawa Y, Sato N, Umezawa K, Simizu S (2015). Conophylline protects cells in cellular models of neurodegenerative diseases by inducing mammalian target of rapamycin (mTOR)-independent autophagy. J BIOL CHEM.

[49] Jang W, Kim HJ, Li H, Jo KD, Lee MK, Yang HO (2015). The Neuroprotective Effect of Erythropoietin on Rotenone-Induced Neurotoxicity in SH-SY5Y Cells Through the Induction of Autophagy. MOL NEUROBIOL.

[50] Liu J, Bi Y, Luo R, Wu X (2011). Simultaneous UFLC-ESI-MS/MS determination of piperine and piperlonguminine in rat plasma after oral administration of alkaloids from Piper longum L. : application to pharmacokinetic studies in rats. J Chromatogr B Analyt Technol Biomed Life Sci.

[51] DUNHAM NW, MIYA TS (1957). A note on a simple apparatus for detecting neurological deficit in rats and mice. J Am Pharm Assoc Am Pharm Assoc.

[52] Ogawa N, Hirose Y, Ohara S, Ono T, Watanabe Y (1985). A simple quantitative bradykinesia test in MPTP-treated mice. Res Commun Chem Pathol Pharmacol.

[53] Lu L, Zhao C, Liu Y, Sun X, Duan C, Ji M, Zhao H, Xu Q, Yang H (2005). Therapeutic benefit of TH-engineered mesenchymal stem cells for Parkinson's disease. Brain Res Brain Res Protoc.

[54] Spinazzi M, Casarin A, Pertegato V, Salviati L, Angelini C (2012). Assessment of mitochondrial respiratory chain enzymatic activities on tissues and cultured cells. NAT PROTOC.

[55] Yang W, Wang X, Duan C, Lu L, Yang H (2013). Alpha-synuclein overexpression increases phospho-protein phosphatase 2A levels via formation of calmodulin/Src complex. NEUROCHEM INT.

[56] Du TT, Wang L, Duan CL, Lu LL, Zhang JL, Gao G, Qiu XB, Wang XM, Yang H (2015). GBA deficiency promotes SNCA/alpha-synuclein accumulation through autophagic inhibition by inactivated PPP2A. AUTOPHAGY.

